# 20 Years of Olympic Media Research: Trends and Future Directions

**DOI:** 10.3389/fspor.2020.572495

**Published:** 2020-09-29

**Authors:** Andrea N. Geurin, Michael L. Naraine

**Affiliations:** ^1^Institute for Sport Business, Loughborough University London, London, United Kingdom; ^2^Faculty of Applied Health Sciences, Brock University, St. Catharines, ON, Canada

**Keywords:** Olympic Games, media, systematic literature review (SLR), communication, sport management, sport media, sport communication

## Abstract

The Olympic Games is the largest multisport event in the world, regularly drawing global audiences in the billions to watch coverage of athletes from hundreds of nations. It has received a great deal of scholarly attention, especially in terms of media coverage, consumption, and co-creation. As coverage has the ability to impact media consumers' perceptions of nations, cultures, and issues, it is important to develop an understanding of research trends relating to the Olympic Games and media in order to uncover gaps in the literature which may be filled by future scholarly work. Therefore, in order to highlight trends in the established literature and uncover areas for development, a systematic literature review was conducted to examine the state of Olympic media research over a 20-year time period (1999–2018). A total of 221 articles were examined, revealing insights into the types of research being produced from theoretical, methodological, and contextual perspectives. Results revealed a significant proportion of scholarship focused on the Summer Olympic Games, the United States, newspaper accounts of the Games, and utilized media framing and agenda setting frameworks and the content analysis methodology. Just over half of the studies utilized a theoretical or conceptual framework, the prevalence of which increased over time. Core areas for continued development in the Olympic media space include embracing and grounding research in theory, diversification in research context, and expanding upon the definition of the Olympic Games within the greater Olympic Movement.

## Introduction

Since its inception in 1896, the modern Olympic Games have grown to become the largest multisport event in the world. The last two editions of the Games, held in PyeongChang, South Korea in 2018, and Rio de Janeiro, Brazil in 2016, featured 2,833 athletes from 92 nations and 11,238 athletes from 207 nations, respectively (PyeongChang 2018, 2020; Rio 2016, 2020). While a very small portion of the worldwide population is able to attend the Games in person, media coverage of the Games reaches global audiences of at least 4.5 billion [International Olympic Committee (IOC), 2019]. It is well-documented in the Olympic media literature that coverage of the Games has the ability to impact entire nations and cultures, and therefore the study of such media is important. For example, Eagleman et al. (2014) stated that media coverage plays a large role in crafting the reality of the Olympic Games for those who cannot attend and experience it firsthand, and the ways in which media outlets present the Olympic Games can have “profound impacts on citizens' views of topics such as gender, nationality, and the perceived importance of some sports over others” (p. 457).

The widespread popularity and increased use of social media since the 2004 Summer Olympic Games means that today's global audiences are not just consuming media coverage about the Games, but are actively contributing to it and engaging with it as well (Liu, 2016), therefore participating in reality co-creation of the Games along with traditional media sources. During the 2018 PyeongChang Winter Olympics, over 1.6 billion Olympic-related videos were viewed on social media platforms such as Facebook, Twitter, Instagram, SnapChat, and YouTube (Publicis Media, 2018). Despite the prevalence of social media usage to consume the Olympic Games (along with traditional consumption methods such as television or newspaper), two comprehensive literature reviews of social media and sport both uncovered only one study relating to the Olympic Games up to the year 2015 (Abeza et al., 2015; Filo et al., 2015).

The study of media in the form of coverage, consumption, and co-creation during the Olympic Games is a popular research stream within the sport management literature, yet no large-scale review of this literature exists. Given the importance of Olympic media coverage in presenting the reality of the event for the billions of sport consumers who cannot attend the Games in person, combined with the more recent developments of social media consumption and usage relating to the Olympic Games, a review of literature related to Olympic media is necessary in order to reveal what is being studied about this topic. For example, what forms of media have been most widely studied? From what national contexts? Using which methods and theories? Answering questions such as these through a detailed review of relevant literature is important to help Olympic media scholars understand where gaps exist in this line of inquiry in order to better focus research in under-developed areas and to replicate past studies where necessary. Schulenkorf et al. (2016) stated that, “review articles are important as they examine and summarize past research by drawing comprehensive conclusions from many separate studies that address a similar topic” (p. 23). Therefore, the purpose of this review was to examine the state of Olympic media research through the use of a Systematic Literature Review (SLR). Doing so will ensure that a wide range of topics and contexts relating to Olympic media are studied in the future to ensure that a well-rounded, highly informative body of literature exists for scholars, students, and practitioners.

The following research questions (RQs) were developed to guide this study, all within the context of research published over a 20-year time period between 1999 and 2018:

RQ_1_: What Olympic contexts (i.e., specific Olympic Games) were examined?
RQ_2_: What specific geographic contexts (i.e., countries) were examined under the lens of Olympic media?
RQ_3_: What forms of media (e.g., newspaper, magazine, television, radio, social media, etc.) were examined in studies focused on Olympic media?
RQ_4_: What research methods were utilized to study the topic of Olympic media?
RQ_5_: What theoretical frameworks were utilized in studies focused on Olympic media?
RQ_6_: In which academic journals were studies relating to Olympic media published?

The following section describes the method used to conduct this study in greater detail.

## Materials and Methods

This paper followed a Systematic Literature Review (SLR) method, which aims to “collect, sum up and evaluate evidence about a particular area” (Ahmed et al., 2019, p. 75). Ahmed et al. (2019) also noted that SLR can expose gaps in the literature on a specific topic, allowing for deeper insight on the topic at hand, as well as bringing to light any recommendations for future research. According to Snyder (2019), literature reviews “have the capacity to engender new ideas and directions for a particular field” (p. 339). SLR has been utilized in research from several different disciplines to study topics such as social media use for knowledge sharing (e.g., Ahmed et al., 2019), software engineering (e.g., Kitchenham et al., 2009), and screen time and sleep for children (e.g., Hale and Guan, 2015). SLR is considered by many to be the gold standard for literature reviews, and the number of business-related literature reviews using the SLR method is growing (Snyder, 2019).

Kitchenham (2004) presented a seven-step process for conducting an SLR, consisting of the following: (1) identifying the need for a review; (2) developing a review protocol; (3) identifying the research; (4) selecting the primary studies; (5) performing a quality assessment; (6) extracting and monitoring the data; and (7) synthesizing the data. Snyder (2019) largely echoed this advice through four steps to design, conduct, analyze, and write the review. For the current study, the need for a review of literature relating to Olympic media was established in the introduction section; however, a review of the additional six steps from Kitchenham's process occurs here.

The next step, developing a review protocol, reduces the chance for research bias by developing specific methods for selecting the literature to be reviewed in the SLR (Kitchenham, 2004). It allows the researchers to set specific criteria for study selection as well as to determine how data will be collected and organized. One step in the review protocol process is to establish inclusion and exclusion criteria for the research studies to be examined. Inclusion and exclusion criteria ensure “that all selected primary studies in the SLR are pertinent, and are related to the study” (Ahmed et al., 2019, p. 75). The inclusion criteria employed for this study included studies:

1) relating to both the Olympic Games *and* media,
2) published in peer-reviewed journals,
3) published between 1999 and 2018,
4) written in English, and
5) available in full-text format in the lead researcher's University library database (a top-25 global University according to the Center for World University Rankings) *or* through an alternate option such as the author's institutional website, directly from the journal website, or another website (e.g., ResearchGate or Academia.edu).

Conversely, the exclusion criteria for this study included studies that:

1) were not related to both the Olympic Games *and* media,
2) were not published in peer-reviewed journals,
3) appeared outside the search timeframe (1999 through 2018),
4) were not written in English,
5) were not available in full-text format from the lead researcher's University library database *or* other websites as detailed in the inclusion criteria.
6) duplicate studies (those that appeared multiple times in the search results).

The choice to examine research from a 20-year time period was made, as this was thought to be most relevant to understanding more recent trends in Olympic media research. With the 2008 Beijing Olympic Games being the first in which social media/Internet use was prominent (Hutchins and Mikosza, 2010), the time period of this study provided us with 10 years before social media and Internet usage was commonplace and 10 years after it became so.

After the inclusion and exclusion criteria were set, the next step was to collect articles that met the search criteria. When deciding how to search for the articles, we looked to two highly-cited SLRs focused on social media and sport. Filo et al. (2015) conducted a manual search of 12 specific sport management-focused journals, uncovering 70 articles for examination, whereas Abeza et al. (2015) conducted searches using five online databases, resulting in 123 articles for examination. Based on these two different search strategies, we decided to adopt the both strategies in an attempt to maximize the number of articles we could examine. First, the researchers used Abeza et al. (2015) database method. EBSCO Discovery, a database from the lead researcher's University library, allowed for 72 different databases to be included in the search using the keywords “Olympic” and “media.” The popular SportDiscus database was not included in these, so it was searched separately. Using features of the databases, the researchers were able to limit searches to peer-reviewed journal articles published during the years 1999–2018, and were able to limit the searches to only those studies published in English.

A total of 173 articles from the database search met the inclusion criteria and passed a brief quality assessment to determine whether each article met the inclusion criteria of the study. Additionally, the following questions were posed about each article that fit the inclusion criteria:

1. Is the topic addressed in the paper focused specifically on the topic of media in the context of the Olympic Games?
2. Is it clear that this is a research study, and not another type of article published in a peer-reviewed journal, such as an introduction to a special issue or a book review?

The 173 articles resulting from the database search well-exceeded the SLRs focused on sport and social media conducted by Abeza et al. (2015) and Filo et al. (2015). Articles were downloaded in their full-text version and saved in a secure folder. Several duplicate articles appeared in the results, and only the first appearance of an article was collected.

Next, the authors adopted the search strategy utilized by Filo et al. (2015) and employed a systematic manual search, which Teare and Taks (2020) recommended for increasing the rigor of a systematic literature review. This search involved the researchers visiting the websites of every journal that appeared in the list of articles and manually searching the journal sites for articles that did not appear in the database search (Teare and Taks, 2020). The researchers viewed the table of contents for every issue of the journals from 1999 to 2018. When potentially relevant articles were discovered based on the article title, the full-text article was downloaded in order to determine whether it met the study's criteria. If so, it was saved in the secure folder with the other articles. The researchers also visited the website for *Journal of Sports Media*, a popular sport communication journal that was not included in any of the databases. Following this manual search, the total number of articles reached 221.

### Coding Procedures

A coding protocol including definitions of each variable was developed to guide the coding process, and a data extraction form was developed to allow the researchers to record all necessary information from the articles. The following variables were adopted from similar studies by Abeza et al. (2015), Filo et al. (2015), and Schulenkorf et al. (2016) and were coded in a Microsoft Excel spreadsheet for each article: study ID (assigned number for each study), study title, author name(s), journal name, publication year, theoretical/conceptual framework, Olympic context (e.g., 2016 Rio Summer Olympic Games), geographic context, media format(s) examined/focused on in the study, method, purpose statement, and DOI. Some studies focused on a specific sport or athlete, and in those instances the sport name or athlete name was also recorded in the “Olympic context” variable.

The lead researcher served as the primary reviewer (data coder) in this study. Fink (2019) indicated that while two reviewers may be used in SLR studies, one reviewer is acceptable. In order to complete the data extraction form, the lead researcher read the abstract, introduction, theoretical/conceptual framework section (if one existed), and the method section of every article in the dataset. When questions arose about a specific code (e.g., theoretical/conceptual framework), the researcher read additional segments of the article in an attempt to find the answer. For example, there were several articles in which it was unclear whether a theoretical or conceptual framework was used. One common example occurred when the name of a framework was used in the title of the article (e.g., “Framing of …”), yet framing theory, an explanation of the term “framing,” and/or a reference to the foundational work on this theory were never mentioned within the article body. Instances such as this required the researcher to re-read the aforementioned sections of the article as well as other parts of the article in order to ascertain whether a theory was actually used.

Following the coding, the lead researcher recoded 10% of the sample in order to test intra-coder reliability, which is recommended by Wimmer and Dominick (2006) when only one coder is used in a study such as this one. All codes were identical between the initial coding and the intra-coder reliability coding, therefore it was determined that the coding was reliable.

See Table 1 for a breakdown of the number of articles published in each year (1999–2018). Based on the findings from this data, the results of our six research questions are presented in the following section.

**Table 1:** Publications per year, 1999–2018.

**Year**	**# Of articles published**	**% Of articles published**
1999	3	1.4
2000	3	1.4
2001	0	0.0
2002	6	2.7
2003	1	0.5
2004	1	0.5
2005	4	1.8
2006	2	0.9
2007	5	2.3
2008	4	1.8
2009	11	5.0
2010	23	10.4
2011	15	6.8
2012	26	11.8
2013	24	10.9
2014	17	7.7
2015	23	10.4
2016	19	8.6
2017	14	6.3
2018	20	9.0

## Results

### Olympic Context

The first research question asked which Olympic contexts were examined in the literature focused on Olympic media from 1999 to 2018. In some instances, studies focused on aspects of the Olympic Games such as the bid phase or legacy. In these instances, the articles were coded according to the Games focused on within the article. Studies that focused on multiple Olympic Games were recoded to accurately indicate the number of studies focused on each Olympics, resulting in 297 cases. Overwhelmingly, the 2008 Beijing Olympic Games and the 2012 London Summer Olympic Games received the most attention, with 18.9 and 18.5% of all articles, respectively. Aside from these two Games, no other edition of the Olympics received more than 7.7% of scholarly attention, with the 2004 Athens Summer Olympic Games receiving this amount. Next, the 2016 Rio Olympics received 6.7% of the scholarly attention, the 2000 Sydney Summer Olympics and 2014 Sochi Winter Olympics both received 5.7%, and the 1996 Atlanta Summer Olympics and 2010 Vancouver Winter Olympics both received 5.1%. Four editions of the Paralympic Games (2008, 2010, 2012, and 2016) were featured in the literature, constituting 2.9% of all coverage. Additionally, two articles focused on the Special Olympics were included, constituting 0.6% of coverage. Table 2 presents the full findings of this research question.

**Table 2:** Scholarly articles from 1999 to 2018 focused on each Olympic Games.

**Olympic Games**	**# Of articles**	**% Of articles**
1924 Paris Summer Games	1	0.3
1928 Amsterdam Summer Games	1	0.3
1936 Berlin Summer Games	4	1.3
1948 London Summer Games	4	1.3
1952 Helsinki Summer Games	3	1.0
1956 Melbourne Summer Games	2	0.7
1960 Rome Summer Games	2	0.7
1964 Tokyo Summer Games	4	1.3
1968 Mexico City Summer Games	5	1.7
1972 Munich Summer Games	2	0.7
1976 Montréal Summer Games	1	0.3
1980 Moscow Summer Games	2	0.7
1984 Los Angeles Summer Games	7	2.4
1988 Seoul Summer Games	7	2.4
1992 Barcelona Summer Games	6	2.0
1994 Lillehammer Winter Games	2	0.7
1996 Atlanta Summer Games	15	5.1
1998 Nagano Winter Games	3	1.0
2000 Sydney Summer Games	17	5.7
2002 Salt Lake City Winter Games	4	1.3
2004 Athens Summer Games	23	7.7
2006 Torino Winter Games	5	1.7
2008 Beijing Summer Games	56	18.9
2008 Beijing Summer Paralympics	1	0.3
2009 Special Olympics Great Britain National Summer Games	1	0.3
2010 Vancouver Winter Games	15	5.1
2010 Vancouver Winter Paralympics	1	0.3
2012 London Summer Games	55	18.5
2012 London Summer Paralympics	6	2.0
2014 Sochi Winter Games	17	5.7
2015 Special Olympics World Games	1	0.3
2016 Rio de Janeiro Summer Games	20	6.7
2016 Rio de Janeiro Summer Paralympics	1	0.3
2018 PyeongChang Winter Games	2	0.7
2020 Tokyo Summer Games	1	0.3

Along with each individual edition of the Olympic Games, we collected data on specific sports that formed the focus of some studies. Of those studies focused on a specific sport during the Summer Olympics, gymnastics was the most popular with nine articles. Next was track and field (*n* = 5), equestrian (*n* = 2), basketball (*n* = 2), soccer (*n* = 2), softball (*n* = 2), and beach volleyball (*n* = 2). Baseball, swimming and triathlon were each the focus of one article. Additionally, one individual athlete, South African Olympian and Paralympian Oscar Pistorius, was the focus of two articles. In terms of the Winter Olympics, five studies focused on ice hockey, four focused on figure skating, and one focused on snowboarding. Those were the only three Winter sports garnering specific focus in the academic literature on media and the Olympic Games.

### Geographic Context

The second research question sought to determine what specific geographic contexts were examined under the lens of Olympic media. In terms of the numbers of countries that each study focused on, the majority (*n* = 147; 66.5%) focused on just one country. Following that, 33 studies (14.9%) focused on two countries, while 20 (9.0%) had no specific country focus. A total of seven studies (3.2%) focused on three countries, four (1.8%) focused on four countries, three (1.4%) focused on six countries, two (0.9%) focused on five countries, and one (0.5%) study focused on 8, 9, 10, 21, and 26 countries.

In terms of which countries were the focal points of these studies, 48 different countries were identified in the 221 articles examined. Because some studies focused on multiple geographic contexts, the data were recoded to accurately indicate the number of times each country was the focal point of a study. The country receiving the greatest attention was the United States (*n* = 97; 27.1%). Following the U.S., China served as the geographic context for 37 articles (10.3%), then the U.K. (*n* = 34; 9.5%), Canada (*n* = 21; 5.9%), Australia (*n* = 20; 5.6%), South Korea (*n* = 13; 3.6%), and Japan (*n* = 12; 3.4%). Russia, Germany, France, and Slovenia were all the focal point of eight articles (2.2%), followed by Brazil with six articles (1.7%), then Spain with five articles (1.4%). Italy, Sweden, and North Korea each served as the geographical context in four articles (1.1%), and Thailand, Switzerland, and South Africa each featured in three articles (0.8%). Several countries were the geographic focus of two articles (0.6% each), including Bulgaria, Colombia, India, Indonesia, Israel, Jamaica, Kenya, Mexico, the Netherlands, Norway, Poland, South Africa, and Taiwan. Finally, the countries serving as the geographical context in just one article (0.3% each) were Algeria, Belgium, Cameroon, Cuba, England (the focus of this article was solely on England–not the greater U.K.), Finland, Greece, Hong Kong, Hungary, Ireland, Kazakhstan, New Zealand, Peru, Saudi Arabia, Singapore, Sri Lanka, and Tibet.

### Media Formats

The third research question asked which forms of media were examined in studies focused on Olympic media. In terms of the number of media formats examined in each study, the vast majority (*n* = 163; 73.8%) examined only one media format. A non-descript number of multiple formats formed the focus of 24 studies (10.9%). These were typically studies in which media consumers were surveyed or interviewed about how media influenced their perceptions of a specific Olympic-related topic/issue, and participants were not asked to specify which media they used to form their opinion. Following that, 21 (9.5%) studies examined two media formats, 10 (4.5%) studied three formats, two (0.6%) studied four media formats, and just one (0.3%) examined five forms of media.

With regard to the specific media formats that were the focus of examination, those studies in the “non-descript number of multiple formats” were removed for further analysis. Of the remaining formats, results revealed that 40.7% (*n* = 96) of studies focused on newspaper content, followed by 32.2% (*n* = 76) focused on television, then websites at 9.7% (*n* = 23), social media at 4.7% (*n* = 17), policy documents at 2.0% (*n* = 7), streaming media at 1.4% (*n* = 5), radio, magazines, and smartphone/mobile at 0.8% (*n* = 3) each, films at 0.6% (*n* = 2), and calendars at 0.3% (*n* = 1). Of the 17 studies focused on social media, six were non-descript in explaining the platform(s) examined, five focused on Twitter, two on Facebook, and one each on Instagram, YouTube, and Weibo. One study indicated that it focused on three different social media platforms: Facebook, Instagram, and Twitter.

**Figure 1:**
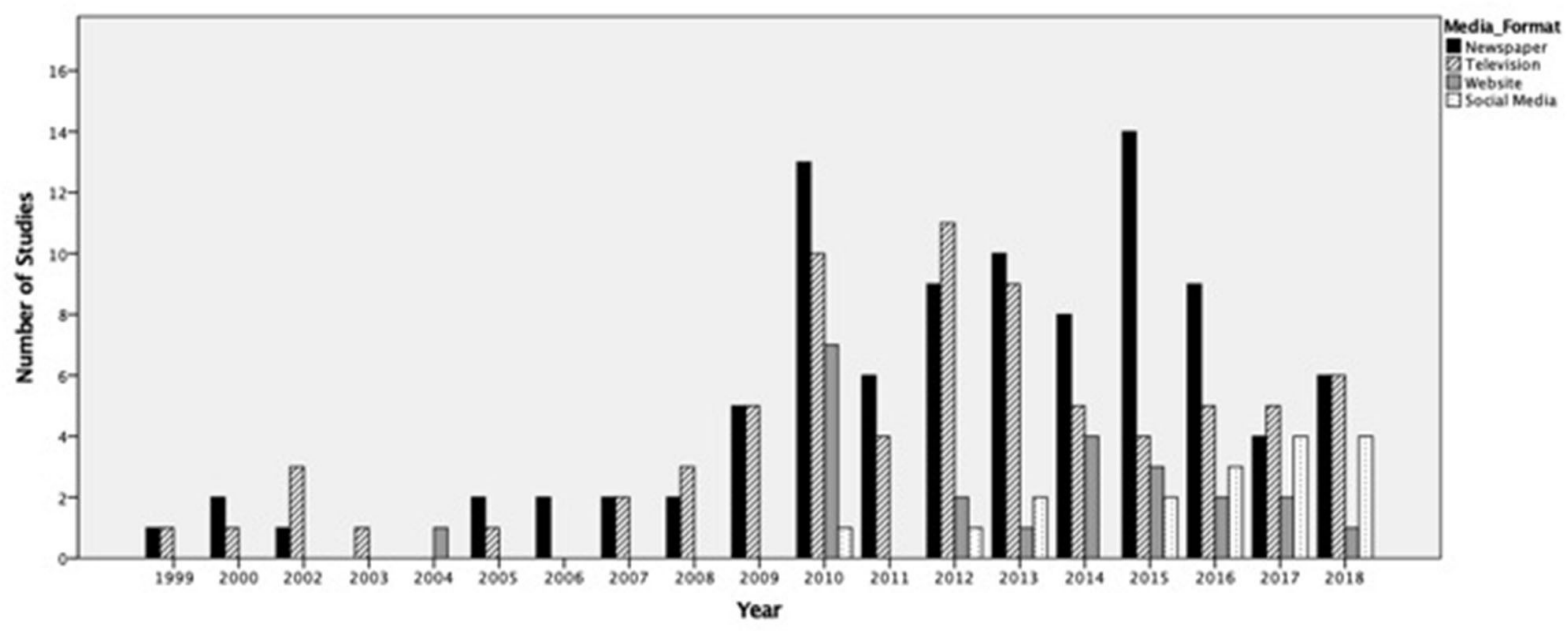
Prevalence of the top four overall media formats studied by year, 1999–2018.

Figure 1 shows the frequency of studying the top four media formats (newspaper, television, websites, and social media) over the time period of the study. Newspaper was by far the most popular medium to study over the duration of the 20 years, as it was the most-studied or tied as the most-studied in 13 of the 19 years (note, there were no studies recorded in 2001). Only television (2002, 2003, 2008, 2012, and 2017) and websites (2004) were the focus of inquiry more frequently than newspaper in the years listed.

### Methods Utilized to Study Olympic Media

The fourth research question sought to determine what research methods were utilized to study Olympic media. In this section, we present data regarding the method(s) used in each study, as well as whether data were analyzed using quantitative, qualitative, or mixed methods. In instances where multiple methods were used (e.g., content analysis and survey), each method was coded separately, resulting in a total of 230 cases. By far, the most popular methodological approach was content analysis, with 76.5% (*n* = 176) of all studies using this method. Of those, 52.3% (*n* = 92) were qualitative (e.g., thematic analysis, discourse analysis, qualitative document analysis), 39.2% (*n* = 69) were quantitative, and 8.5% (*n* = 15) were mixed methods. We acknowledge that the listed qualitative methods approach the analysis of media content in different ways, but we made the decision to group them together due to the fact that, fundamentally, they all qualitatively analyze media content.

The next most popular method was surveys at 10.9% (*n* = 25), then historical analyses with 3.9% (*n* = 9), and interviews with 3.5% (*n* = 8). Ethnography and review articles were both used four times, with each method comprising 1.7% of the sample. Finally, case study was used twice (0.9%), and both observation and experimental design were used once (0.4% each). Overall, the majority of studies used qualitative methods of analysis (51.3%; *n* = 118), with quantitative methods ranked second (40.0%; *n* = 92), and mixed methods a distant third (8.7%; *n* = 20). Table 3 displays the full results of the methodological approaches used.

**Table 3:** Methodological approaches.

	**Quantitative**	**Qualitative**	**Mixed methods**	**Total**
Content analysis	69	92	15	176
Survey	22	1	2	25
Historical analysis	0	9	0	9
Interviews	0	7	1	8
Review	0	3	1	4
Ethnography	0	3	1	4
Case study	0	2	0	2
Experiment	1	0	0	1
Observation	0	1	0	1
Total	92	118	20	230

### Theoretical and Conceptual Frameworks

**Figure 2:**
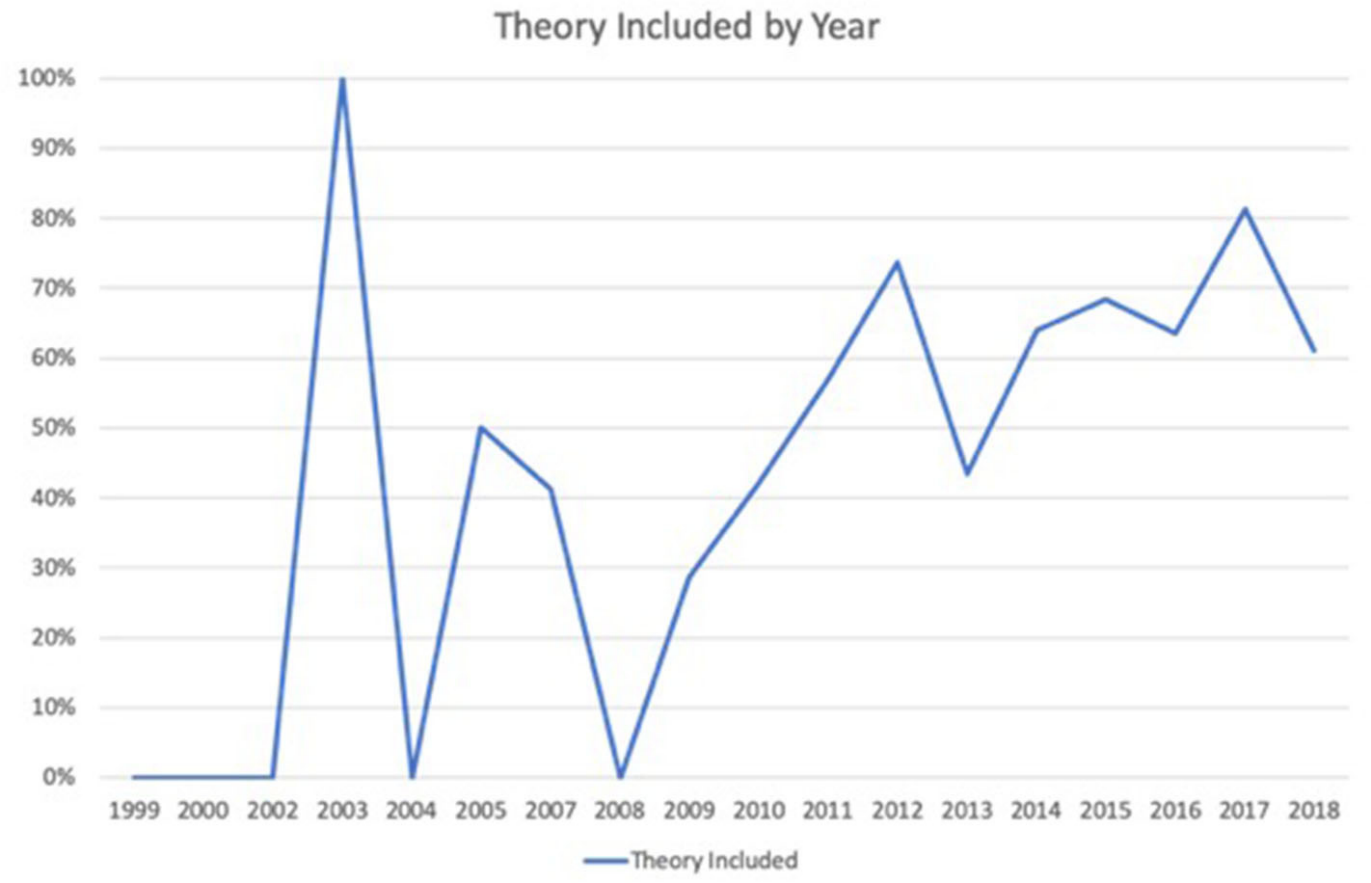
Percentage of publications that included a theory, 1999–2018.

The fifth research question asked what theoretical frameworks were utilized in research studies focused on Olympic media. Results showed that just over half (*n* = 120; 54.3%) of the articles utilized a theoretical or conceptual framework for their research, while 45.7% (*n* = 101) did not. In total, 32 different theories were used, and in some cases, authors developed their own conceptual framework based on different topics in the literature. Table 4 displays the specific theories utilized, while Figure 2 shows the prevalence of utilizing a theory over the time period of the study. With 2003 as an outlier at 100%, the use of a theoretical framework appeared to increase over time. Please note that in instances where multiple theories were used, these were coded separately.

**Table 4:** Theoretical approaches utilized in Olympic media research.

**Theory name**	**# Of articles**	**% Of articles**
Framing	39	26.5
Conceptual framework developed by the study's authors	32	21.8
Agenda setting	17	11.6
Nationalism	7	4.8
Self-categorization	5	3.4
Social identity	5	3.4
Gender	3	2.0
Hegemonic masculinity	3	2.0
Nation branding	3	2.0
Cultivation	3	2.0
Uses and gratifications	3	2.0
Critical feminist	3	2.0
Social cognitive	2	1.4
Self-presentation	2	1.4
Social constructionism	2	1.4
Symbolic boasting	1	0.7
Globalization	1	0.7
Hegemonic femininity	1	0.7
Invented traditions	1	0.7
Competitive identity	1	0.7
Information integration	1	0.7
Theory of the niche	1	0.7
Economic demand	1	0.7
Impression management	1	0.7
Parasocial interaction	1	0.7
Active audience	1	0.7
Structuration	1	0.7
Disposition	1	0.7
Gender power relations	1	0.7
Invented traditions	1	0.7
Mobile communication	1	0.7
Social role	1	0.7
Technology adoption	1	0.7

### Academic Journals

The sixth and final research question asked in which academic journals the studies relating to Olympic media were published. In terms of the scholarly fields in which research was published, the majority (*n* = 134; 60.6%) fell into the category of sport subjects. Following that, 58 articles (26.2%) were published in general media/communication journals, 23 (10.4%) were published in other social science journals, four (1.8%) in event/tourism journals, and two (0.9%) in health/medicine journals.

There were 77 different journals represented in this study. The most popular journal to publish Olympic media-related research was the *International Journal of the History of Sport* with 27 articles (12.2%). Following that was *Sport in Society* with 21 articles (9.5%), *International Review for the Sociology of Sport* and *International Journal of Sport Communication* with 17 articles each (7.7%), and rounding out the top five was *Mass Communication and Society*, the only non-sport specific journal in the top five, with 14 articles (6.3%). Table 5 presents the full figures for all journals represented in this study.

**Table 5:** Journals represented in this study.

**Journal name**	**# Of articles**	**% Of articles**
*International Journal of the History of Sport*	27	12.2
*Sport in Society*	21	9.5
*International Review for the Sociology of Sport*	17	7.7
*International Journal of Sport Communication*	17	7.7
*Mass Communication and Society*	14	6.3
*Journal of Sports Media*	13	5.9
*Communication and Sport*	11	5.0
*Journal of Broadcasting and Electronic Media*	8	3.6
*Journal of Sport and Social Issues*	6	2.7
*Media Culture and Society*	5	2.3
*Journal of Sport Management*	4	1.8
*Sociology of Sport Journal*	3	1.4
*Media International Australia*	2	0.9
*Women in Sport and Physical Activity Journal*	2	0.9
*American Journalism*	2	0.9
*The China Quarterly*	2	0.9
*Convergence: The Journal of Research into New Media Technologies*	2	0.9
*International Journal of Sports Marketing and Sponsorship*	2	0.9
*International Journal of Communication*	2	0.9
*Sociological Research Online*	2	0.9
*Journalism Practice*	2	0.9
*The Journal of International Communication*	2	0.9
*Sport Management Review*	1	0.5
*Journal of Sport and Tourism*	1	0.5
*Asian Studies Review*	1	0.5
*Television and News Media*	1	0.5
*Southeast Review of Asian Studies*	1	0.5
*Journal of Applied Social Psychology*	1	0.5
*Observatorio Journal*	1	0.5
*Journal of Australian Studies*	1	0.5
*International Journal of Event and Festival Management*	1	0.5
*Journal of Sport History*	1	0.5
*Canadian Bulletin of Medical History*	1	0.5
*Journal of International Women's Studies*	1	0.5
*Sociology*	1	0.5
*China Media Research*	1	0.5
*Communication Research*	1	0.5
*International Communication Gazette*	1	0.5
*Journal of Applied Sport Psychology*	1	0.5
*Science of Gymnastics Journal*	1	0.5
*Global Media Journal*	1	0.5
*Media, War and Conflict*	1	0.5
*African Journalism Studies*	1	0.5
*European Journal of Communication*	1	0.5
*The Fibreculture Journal*	1	0.5
*Journal of Popular Television*	1	0.5
*Qualitative Research in Sport, Exercise, and Health*	1	0.5
*International Humanities Studies*	1	0.5
*Journal of Language and Social Psychology*	1	0.5
*Journal of Public Health*	1	0.5
*Journal of Vacation Marketing*	1	0.5
*Interactions: Studies in Communication and Culture*	1	0.5
*Journal of Children and Media*	1	0.5
*Psychology of Sport and Exercise*	1	0.5
*European Journal of Sport Science*	1	0.5
*Journalism and Mass Communication Quarterly*	1	0.5
*Feminist Media Studies*	1	0.5
*Movimento*	1	0.5
*Nordicom Review*	1	0.5
*Journalism Studies*	1	0.5
*Journal of Language and Politics*	1	0.5
*Asian Journal of Communication*	1	0.5
*Online Information Review*	1	0.5
*Digital Creativity*	1	0.5
*Gender and Language*	1	0.5
*Polish Sociological Review*	1	0.5
*Russian Journal of Communication*	1	0.5
*Tourism Management*	1	0.5
*Historical Social Research*	1	0.5
*Journalism*	1	0.5
*Physical Culture and Sport*	1	0.5
*Journal of Sports and Entertainment Law*	1	0.5
*Communication Reports*	1	0.5
*Sustainability*	1	0.5

## Discussion

The purpose of this review was to examine the state of Olympic media research with the intent of highlighting trends in the established literature base, while simultaneously uncovering areas for development. The review yielded insights into the types of research being produced from theoretical, methodological, and contextual perspectives. Based on the results, this section provides further discussion of the following key areas identified for continued development in the Olympic media research space: embracing and grounding research in theory, diversification in research context, and expanding upon the definition of the Olympic Games within the greater Olympic Movement. The section concludes with explanations five recommendations for future research.

### Embracing and Grounding Research in Theory

Given the intersection of sport with cultural, economic, and political elements, it was surprising to find that just over half of the articles from this study utilized a theoretical or conceptual framework. Of those that did, it was encouraging to see that 67 (55.8%) were from the past 5 years. Overall, 67.0% of all articles published within the last 5 years (2014–2018) used a framework of some kind. Still, it is discouraging that one-third of Olympic media research articles published in the last 5 years did not incorporate theory. While there is certainly value in empirical research, and indeed this study is an example of that, the myriad research that exists on the topic of Olympic media would lead one to believe that more of the current literature would build on this body of work, and therefore utilize theoretical and conceptual frameworks. This finding is similar to that of Filo et al. (2015), in which the majority of social media-related sport studies did not incorporate a theoretical framework.

Cunningham (2013) noted that “theory is a bedrock upon which good scholarship rests” (p. 2). Theory is useful because it explains, it guides the creation of research questions, and while theory contributes to our research, our research may also contribute to theory (Doherty, 2013). Creswell (2003) explained that some qualitative studies may not use an existing theory to guide their work, but noted the importance of grounded theory in such studies. Whether utilizing an existing theory or grounded theory, sport management scholars have articulated the importance of theory in our field's research. For example, Irwin and Ryan (2013) explained that research guided by theory should not be separate from research that seeks to inform industry (i.e., applied research), and the two can be combined into a mutually beneficial manner. The notion of using theory to inform applied research and practice is not new, as Earle Zeigler, one of the modern founders of sport studies, touted the benefits of balancing the use of theory in applied research over 30 years ago (Zeigler, 1987).

The present study reveals an opportunity and need for Olympic media researchers to incorporate theory into their work to a greater extent in order to best inform both the academic literature and industry. Doing so will contribute to growth in the field of sport management, contribute to existing theories and the development of new theories, and result in effective practice within sport organizations (Doherty, 2013). In the context of this study, theory can specifically assist in informing media organizations and Olympic sport organizations of best practices within their businesses.

### Diversification in Research Context

Beyond theory, there is certainly scope for greater diversity in terms of context. There is a pre-disposition for Olympic media articles to default to studying the newspaper media format, with over 40% of articles in the study utilizing this form. Newspapers have served as a staple within the mass media industry, but shifting consumption patterns have led to a steady decline in newspaper circulation and readership. The Pew Research Center reported that in 2017, print circulation was down 11% from 2016 (Pew Research Center, 2018). Even as media outlets attempt to transition to digital offerings, the overall picture remains bleak. Accounting for the marginal boost in these firms, circulation still declines 4% year-over-year (Pew Research Center, 2018). Yet, despite the glaring weakening of this media format, Olympic media research continues to favor this medium, with 39.05% (*n* = 41) of all studies in the last 5 years of the study (2014-2018) focused on newspaper. In comparison, newspapers were the focus of 41.38% (*n* = 12) of the studies in the first 10 years of this research (1999–2008), when social media and Internet usage was either non-existent or not nearly as prevalent. Despite the prevalence of new media formats for both reporting and media consumption in the later years of this study, researchers still chose to focus on newspapers to nearly the same degree as the earlier years of the study. In terms of the media format and national context, newspaper was the prominent medium examined for all countries except South Korea and Japan (60.00% focused on television in each country), Sweden and Spain (33.33% focused equally on television, radio, and social media), Thailand, Colombia, and India (100.00% focused on websites), South Africa, Indonesia, and Jamaica (100.00% focused on television), Peru (100.00% focused on policy documents), and Kenya (50.00% focused on television and 50.00% focused on websites). The focus on websites and new media in many of these countries indicates that researchers are beginning to expand their geographical horizons in Olympic media research, though not as much as is necessary to truly understand the reality presented to media consumers in these cultures, which is discussed later in the next section.

Although newspaper reports can answer important questions, particularly in cross-national comparisons, as the role of media and news reporting shifts in the sport landscape, scholars must ask themselves whether analyzing a set of newspaper articles alone can provide a sufficient enough dataset necessary to answer important questions and whether it is capable of driving Olympic media research forward with a meaningful contribution. As more individuals opt to consume their news reporting via television, social media, and short-form pages pushed via mobile applications (apps), it is important to increase the study these forms of media since they appear to be more relevant to today's Olympic media consumers.

Another area for expanding the scope of Olympic media research is moving beyond the current concentration of geographic regions. It should come as no surprise that the United States was the country that received the greatest attention in these articles, given the significant role the country has played in the commercialization and financial sustainability in the Olympic Movement (Barney et al., 2002), as well as the fact that this study only reviewed research written in English. The amount of attention Olympic media research has paid toward the United States over other countries, however, is incongruent with the inherent internationalization of the Olympics. For the U.S. to serve as the geographic context nearly eight times as much as Japan and South Korea, and roughly 12 times as much as Russia, Germany, and France ignores what is taking place in other nations (e.g., what reality is being presented to consumers outside of the U.S.? What conversations are taking place on social media about the Olympics outside of the U.S.?) The obvious case for the lack of geographic diversity is the languages spoken and written in those countries, and the inability to translate for English speaking sport researchers. While this is a notable concern, it does not account for the low rates of usage for other English-speaking nations such as Australia and Canada, who were collectively cited in 11.5% of the dataset. Additionally, New Zealand was featured in just one article over the 20-year period, which is surprising given the nation's Summer Olympic success, notably finishing fourth in the “medals per capita” category against all other nations in the world in both 2012 and 2016 [High Performance Sport New Zealand (HPSNZ), 2016].

While the United States has a large number of sport management academic programs, this does not preclude researchers from the U.S. (or researchers from other countries) from looking outside their geographic boundaries for Olympic-related media issues. Naturally, the hesitation for English-only speaking researchers is the inability to analyze and code foreign languages, but this is not always the case. For instance, while Hindi is observed as one of the most common languages in India, English has emerged as the de facto lingua franca. Outlets such as the *Hindustan Times* have reported on India's upcoming Olympic hosting aspirations (Hindustan Times, 2017), and the outlet is entirely in English. Yet, India was the featured geographic region only twice in this SLR. The Americentrism found in these works does not adequately portray the reality of the Olympic Games, which is highly international in scope, as evidenced by the fact that 204 nations participated in the most recent Summer Olympic Games in Rio de Janeiro. While there could be more research currently in press from the 2016 and 2018 Olympic Games, the current skew of articles toward the U.S. is a missed opportunity to better understand Olympic media coverage and issues relating to media in other parts of the world. As Eagleman et al. (2014) explained in their examination of Olympic media coverage from six different nations, a country's size, political structure, culture and history can all contribute to differing presentations of the Olympic Games. Until research adopts a more global focus, however, those differences will not be well-understood.

### Expanding Upon the Definition of the Olympic Games Within the Greater Olympic Movement

The articles examined through the SLR also held a unique, but shared trait: while the various works were about the Olympic Movement, they were pre-dominantly specific to Summer and Winter Olympic Games, with over 82.8% of articles collected featuring the Summer Games, 13.5% focused on the Winter Games, 3% on Paralympic Games, and 0.7% on the Special Olympics. This is problematic for several reasons.

While the Summer Olympic Games outnumber the Winter version in terms of number of countries participating and total number of athletes competing, they are still an Olympic Games that occur every 4 years with similar issues attached (e.g., cost overruns of facilities, readiness and preparedness, housing and social problems). The upcoming 2020 Tokyo Olympic Games (to take place in 2021) will feature 339 events across 33 sports, while the most recent Winter Olympic Games in PyeongChang in 2018 featured 109 events across 15 disciplines (You're in!, 2016; Winter Olympics full schedule, 2018). The 2018 Winter Games were viewed by 2.6 billion people through 106 Olympic broadcast partners compared to the 2016 Summer Games, which were viewed by 4.5 billion people through 79 broadcast organizations [Olympic Broadcasting Services. (n.d.).]. Despite the Winter Games being roughly one-third the size of the Summer Games in terms of its number of events, it is clearly still a mega-event drawing in billions of viewers from around the globe, and therefore seems worthy of scholarly inquiry related to media coverage. What is especially troubling about this discrepancy between the Summer and Winter Games focus is that the first Winter Olympic Games noted in the SLR was 1994 in Lillehammer, Norway. Although Summer Olympic Games as far back as Paris in 1924 were utilized, 51 total articles focused on Summer Games that occurred prior to Lillehammer and therefore did not see benefit in looking beyond this Winter Games. This includes omitting the “Miracle on Ice” and the first official participation in the Olympic Movement for the People's Republic of China in the 1980 Lake Placid Winter Games, and the qualification of the Jamaican bobsled team or influence of “Eddie the Eagle,” the flamboyant British ski jumper, from the 1988 Calgary Winter Games. As articles retrieved from the SLR were from a recent period, the rationale for these omissions would be clearer (given they are not as timely). However, when scholarship continues to find merit in discussing Games as far back as 1924, it raises doubts about *when* (and *where*) Olympic media research is choosing to set its gaze.

These doubts also exist for Olympic Movement research beyond both the Summer and Winter Olympic Games. Only nine studies focused on the Paralympic Games, two on the Special Olympics, and none on the Youth Olympic Games (YOG). This would suggest that scholarship has purposefully chosen to ignore these events, which is a significant oversight. As the Paralympic Games are held in conjunction with the Olympic Games, they bear importance to the Olympic Movement. Moreover, these events are fraught with their own set of challenges, and media awareness issues. Concurrently, the absence of the YOG and the scant literature on Special Olympics from the SLR is incredibly concerning. Though the Special Olympics are not organized by the IOC, they are officially recognized by the Olympic Movement, and are able to use the word “Olympic” in their title.

The circumstances for the YOG are arguably far more egregious. While there have only been five YOGs to date (i.e., 2010 Singapore Summer YOG, 2012 Innsbruck Winter YOG, 2014 Nanjing Summer YOG, 2016 Lillehammer Winter YOG, and 2018 Buenos Aires Summer YOG), the event is owned by the IOC and can be considered a full-fledged Olympic Games. The Opening Ceremony for the most recent YOG in Buenos Aires drew an estimated 200,000–215,000 live attendants, far eclipsing the numbers for Opening Ceremonies of the Summer or Winter Olympic Games (Youth Olympic Passes Run Out, 2018). In comparison, ~75,000 people attended the Rio 2016 Opening Ceremony (Lackey, 2016), and nearly 35,000 attended the PyeongChang 2018 Opening Ceremony (Calfas, 2018). Additionally, 71 broadcast organizations delivered content from the 28 sports at the 2018 Buenos Aires YOG to 220 territories around the globe, highlighting the expanding profile of this event [Olympic Broadcasting Services. (n.d.).]. Despite a growing number of YOG focused articles, the number of media related scholarship dedicated to YOG is low (cf. Houlihan et al., 2017). What exacerbates the lack of YOG and media research is the rapid change of Olympic consumption by younger audiences (Nielsen Company, 2010). If audience consumption is changing, and new Olympic formats are being introduced (e.g., YOG), then it would be also be logical to address these new formats from a media consumption perspective. Indeed, what these omissions indicate is that the current definition of the Olympic Games in Olympic media research has been quite limited, and is ripe for expansion.

While this study uncovered many insights into the Olympic media research conducted over the past 20 years, this section highlighted the most important findings from a research perspective, relating to embracing and grounding research in theory, diversification in research context, and expanding upon the definition of the Olympic Games within the greater Olympic Movement. The following section identifies key future directions that Olympic media researchers should consider in their pursuits.

### Future Directions

Given these findings, Olympic media research lacks a level of rigor and diversity in its approach. This is not to suggest that the research that has been completed previously is inappropriate or invalid, but there are other areas that can be explored to expand our understanding of media in the Olympic Movement. As such, we make the following recommendations:

1) Researchers should expand their scope beyond media coverage, and highlight the human elements of Olympic media through the use of methods that move beyond simple content analysis.

With 76.5% of all research being conducted via content analysis and just 14.4% utilizing surveys or interviews, it is clear that media consumers, content creators (e.g., journalists, social media users), IOC and organizing committee communication, and, of course, athletes (e.g., their use of social media during the Olympic Games, their relationship with journalists and other digital stakeholders) are largely missing from the current literature. Some scholars have broached these issues from a national sport organization perspective, many of whom govern Olympic sports (e.g., Eagleman, 2013; Naraine and Parent, 2016), but there is scope for continued growth in this space. Indeed, examining media content from the Olympic Movement is relevant, but scholars should also be concerned with the management of Olympic media; placing emphasis on different stakeholder groups as the focal point of inquiry can move Olympic media research beyond a descriptive account toward in-depth accounts with findings beneficial for managers and theorists working and studying in those additional spaces. Borrowing from Wenner (2014) sentiment, content analyses are useful in developing a first-level understanding, but scholars must seek to advance to a new stage that incorporates new methods to provide meaningful contributions. Methods such as surveys, interviews, focus groups, observations, ethnographies, autoethnographies, and netnographies can uncover deeper meanings and different perspectives of Olympic media content.

2) Olympic media research requires significant improvement in the digital space.

For example, researchers could conduct narrative analyses on the video content produced and housed on the Olympic Channel, segment social media users in the Twitter network of multiple organizing committees for similarities, and even perform autoethnographies on navigating Olympic mobile apps and the associated push notifications they might receive. Present scholarship is well-behind in these advancements, and new undertakings are necessary to further understand the digital elements of the Olympic Movement.

3) Scholarship should extend its focus.

This point refers to the geographic region in which content derives, the Olympic Games chosen for study, and media discussions pertaining to the YOG, Paralympics, and Special Olympics. Diversification of geographic region can be achieved via cross-national comparatives; by understanding how multiple jurisdictions respond to one issue, scholars are able to glean the impacts of culture and nationhood on media consumption. Further, scholarship should continue to diversify the Olympic Games contexts chosen. There are Olympic media discussions of value in the Winter Olympic Games, whether in a North American (e.g., Vancouver), European (e.g., Torino), or Asian (e.g., PyeongChang) context. This point is also underscored by scheduled hosts for the next two Winter Olympic Games: Beijing, China, and Milan-Cortina, Italy. Additionally, media representations of events such as the YOG, Paralympics, and Special Olympics, and the extent to which media are involved in these events can have lasting sociological impacts on issues such as youth sport interest, participation, and associated consumption (e.g., social media), and the perceptions of, and, destigmatization of, people with mental and physical disabilities.

4) It is critical for future scholarship to develop current and longitudinal research projects.

Much of the research found in this SLR were analyses based on historical data (28.05% of all articles), which chose to shed light on issues from early editions of the Olympics when sport scholarship would have been limited. However, while the future agenda for Olympic research may include these types of expositions, greater weight needs to be placed on recent events given the new era in the sporting landscape (cf. Parent et al., 2018).

Additionally, future inquiries need to become more aware of the limited contribution a study of or from a single, one-off event can yield. Certainly, delimiting works to a specific event within the Olympic Movement is necessary to set and frame a particular narrative, but there is much room to advance the robustness of Olympic media research through longitudinal approaches. Gathering data across multiple Games in succession, events that are held in the same continent, or even events that are held in the same city are ripe for scholarly pursuits.

It is also prudent for scholars to consider cross-examining the traditional Summer and Winter Olympic Games with their Paralympic counterparts, as well as the YOG and Special Olympics. For instance, the most recent YOG was held in 2018 in Buenos Aires, Argentina, and the operational issues there may have been contextually similar to those of the 2016 Summer Olympic and Paralympic Games in Rio de Janeiro, leaving scope for examinations of media reporting, media management, and social media consumption.

## Conclusion

Utilizing an SLR approach, this study sought to examine the state of Olympic media research over the past two decades to assist in revealing gaps and opportunities for future research. There are some strong positive takeaways from this study. It appears that recent Olympic media research is increasingly embracing theory, reaching levels that were seen more than a decade ago. There is much work, however, that can be done in this and other elements of research on media in the Olympic Movement. Scholars in this space should heed the recommendations of this study to ascertain findings related to pertinent, contemporary issues, as well as expanding the geographical context in which those issues manifest. The Olympic Games are a global sport institution, and thus reseach on this institution should also be global in scope. What we have gleaned over the last 20 years of Olympic media research, however, is a tendency to focus on some contexts over others, leaving gaps and impacting the ability to examine changes from one Games to the next.

It is important to acknowledge the limitations of this study. First, the SLR featured English-language only journals, which had the potential to skew the data in favor of studies conducted in English-speaking nations. We did not attempt to match the authorship teams' national affiliation(s) with the Olympic geographic context that was studied, which future researchers could attempt to analyze. Additionally, there is a possibility that our search strategies did not yield all articles that exist on media and the Olympic Games, which serves as another limitation. Still, a robust total of 221 articles were analyzed, yielding new insights that will assist Olympic media scholars in determining new directions for their research. In expanding research contexts, methodologies, and theoretical approaches, researchers have the opportunity to broaden the scope of research conducted in this area and bring to light new and important knowledge on this topic.

## Author Contributions

AG contributed to the study design, data collection, data coding, and data analysis, as well as the manuscript conceptualization and preparation. MN contributed to the study design, interpretation of results, manuscript conceptualization and preparation, and proofreading. All authors contributed to the article and approved the submitted version.

## Conflict of Interest

The authors declare that the research was conducted in the absence of any commercial or financial relationships that could be construed as a potential conflict of interest.
